# Health insurance and neighborhood poverty as mediators of racial disparities in advanced disease stage at diagnosis and nonreceipt of surgery for women with breast cancer

**DOI:** 10.1002/cam4.6127

**Published:** 2023-06-06

**Authors:** Robert B. Hines, Xiang Zhu, Eunkyung Lee, Bradley Eames, Karolina Chmielewska, Asal M. Johnson

**Affiliations:** ^1^ Department of Population Health Sciences University of Central Florida College of Medicine Orlando Florida USA; ^2^ Research Administration ‐ Operations University of Central Florida College of Medicine Orlando Florida USA; ^3^ Department of Health Sciences College of Health Professions and Sciences University of Central Florida Orlando Florida USA; ^4^ Department of Medical Education University of Central Florida College of Medicine Orlando Florida USA; ^5^ Department of Environmental Sciences and Studies Public Health Program, Stetson University DeLand Florida USA

**Keywords:** breast cancer, epidemiology, registries, screening, surgery, statistical methods

## Abstract

**Background:**

In our recent study, advanced disease stage and nonreceipt of surgery were the most important mediators of the racial disparity in breast cancer survival. The purpose of this study was to quantify the racial disparity in these two intermediate outcomes and investigate mediation by the more proximal mediators of insurance status and neighborhood poverty.

**Methods:**

This was a cross‐sectional study of non‐Hispanic Black and non‐Hispanic White women diagnosed with first primary invasive breast cancer in Florida between 2004 and 2015. Log‐binomial regression was used to obtain prevalence ratios (PR) with 95% confidence intervals (CIs). Multiple mediation analysis was used to assess the role of having Medicaid/being uninsured and living in high‐poverty neighborhoods on the race effect.

**Results:**

There were 101,872 women in the study (87.0% White, 13.0% Black). Black women were 55% more likely to be diagnosed with advanced disease stage at diagnosis (PR, 1.55; 95% CI, 1.50–1.60) and nearly twofold more likely to not receive surgery (PR, 1.97; 95% CI, 1.90–2.04). Insurance status and neighborhood poverty explained 17.6% and 5.3% of the racial disparity in advanced disease stage at diagnosis, respectively; 64.3% remained unexplained. For nonreceipt of surgery, insurance status explained 6.8% while neighborhood poverty explained 3.2%; 52.1% was unexplained.

**Conclusions:**

Insurance status and neighborhood poverty were significant mediators of the racial disparity in advanced disease stage at diagnosis with a smaller impact on nonreceipt of surgery. However, interventions designed to improve breast cancer screening and receipt of high‐quality cancer treatment must address additional barriers for Black women with breast cancer.

## Graphical abstract

In this study, we sought to quantify the magnitude of the Black versus White disparity in advanced disease stage at diagnosis and nonreceipt of surgery for women with breast cancer in Florida. We then used multiple mediation analysis to evaluate potential mediation of the race effect by insurance status and neighborhood poverty.

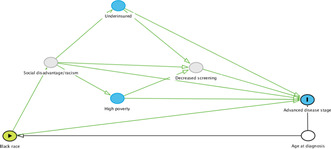

## 1 INTRODUCTION

In the United States (US), breast cancer mortality in non‐Hispanic Black or African American (Black) women has been higher than that of non‐Hispanic White (White) women since 1981. This trend has occurred despite White women having a higher incidence of the disease during this same period.^1^ Many studies and statistical reports have documented racial (Black/White) disparities in breast cancer mortality/survival over several decades.^2^, ^3^, ^4^, ^5^ Despite knowledge of racial disparities in many cancer outcomes and corresponding efforts to redress these inequities, Black women still bear a significantly higher burden of breast cancer than White women.^6^, ^7^ We recently reported that although breast cancer survival had improved for all women in Florida and Black/White disparities had decreased, Black women still experienced over twice the rate of breast cancer death compared to their White counterparts in the most recent time period.^8^ Thus, despite awareness and efforts to mitigate disparities in breast cancer outcomes by race over the past several decades, progress has been slow.

Mediation analysis offers a way for researchers to gain insight into how or why a particular exposure impacts an outcome by decomposing the effect of exposure due to mediators and confounders, that is, third variable effects.^9^, ^10^ By constructing a directed acyclic graph (DAG) or causal diagram hypothesizing the mechanisms through which an exposure affects an outcome, investigators can then use mediation analysis to evaluate the accuracy of their DAGs to ascertain the importance of potential mediators. Thus, the results of these studies can potentially offer high value by identifying important factors which underlie causal mechanisms. Acting on the results of mediation analysis represents an evidence‐based approach to guide interventions, craft health policies, and develop public health programs to eliminate health disparities.

We recently conducted a multiple mediation analysis to determine the most important mediators, at the time of diagnosis and treatment of breast cancer, that explain the racial disparity in 5‐year breast cancer survival for women in Florida. As reported, advanced disease stage at diagnosis and nonreceipt of surgery were the most important mediators contributing to the poorer survival in Black women.^11^ The purpose of the current study was to provide new knowledge concerning the causes of racial disparities in intermediate outcomes along the breast cancer continuum. To achieve this goal, we first quantified the magnitude of the racial disparity for the intermediate outcomes of advanced disease stage at diagnosis and nonreceipt of surgery. Next, we evaluated mediation by the more proximal mediators of insurance status and neighborhood poverty on these two intermediate outcomes in the causal path from race to breast cancer survival. This new knowledge can provide greater insight into racial disparities in breast cancer outcomes and potentially identify more proximal targets for interventions, health care policies, and public health programs.

## 2 METHODS

Women who were residents of Florida, diagnosed with first primary invasive breast cancer between 2004 and 2015, and reported to the Florida Cancer Data System (FCDS) were eligible for this study. Initial study exclusions in our larger study have been described previously.^12^ Additional exclusions included: Hispanic ethnicity (*n* = 16,706), survival <120 days following diagnosis (*n* = 9430), and unknown American Joint Committee on Cancer (AJCC, 6th/7th Edition) disease stage (*n* = 1488). This study was approved by the Institutional Review Boards of the University of Central Florida and the Florida Department of Health.

Data contained in the FCDS include demographic, tumor, treatment, follow‐up, and cause of death information for all cases in the registry. Based on self‐reported race/ethnicity information obtained from the medical record and reported to the FCDS, race was categorized as non‐Hispanic Black or White. The outcomes of interest were AJCC advanced disease stage at diagnosis (Stage III/IV) and nonreceipt of surgery. Based on recommendations regarding timing of cancer‐directed surgery and adding a 30‐day grace period, receipt of surgery was denoted as “yes” if the date of surgery was within 120 days of diagnosis, “no” otherwise.^13^ To ensure that all patients survived long enough to receive treatment, all women were required to have at least 120 days of follow‐up from the date of diagnosis. Consistent with prior studies, insurance status was dichotomized to represent those with Medicaid or those who were uninsured (yes/no) versus those with other forms of health insurance.^14^, ^15^ As Medicaid enrollment is a surrogate for individual‐level poverty, women in the Medicaid group included women who had Medicare but were Medicaid (dual) eligible. The percentage of households in a census tract below the poverty level was obtained from the American Community Survey. Neighborhood (census‐tract) poverty was classified as low (0.0%–4.9%), lower‐middle (5.0%–9.9%), upper‐middle (10.0%–19.9%), and high (≥ 20%) according to percentage of households living in poverty.^16^, ^17^ Neighborhood poverty was also treated as a binary variable and categorized as high poverty (yes/no).

**FIGURE 1:**
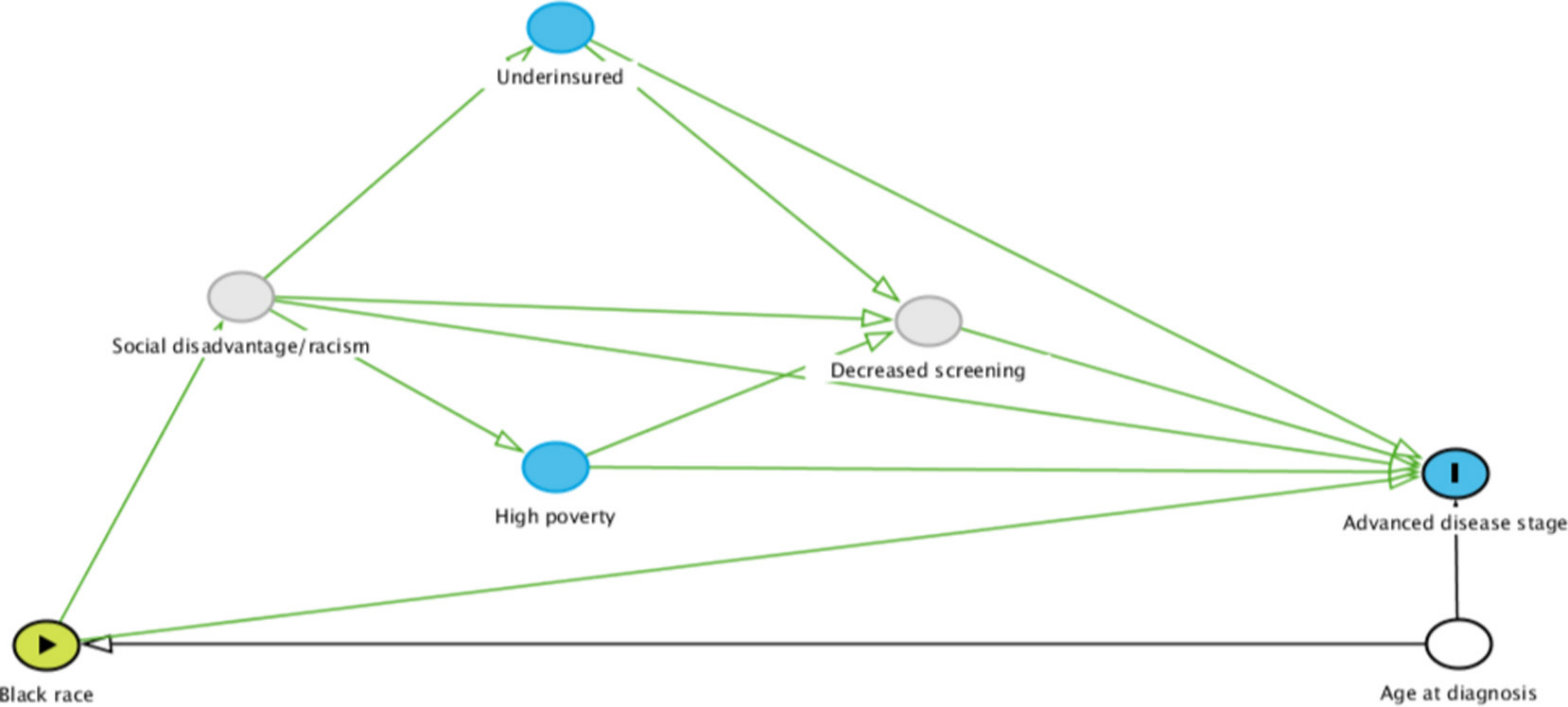
Directed acyclic graph of racial disparity in advanced disease stage at diagnosis. Hypothesized mediators include social disadvantage/racism, underinsurance, high neighborhood poverty, and decreased screening. Gray mediators represent unmeasured data. Age at diagnosis is considered a confounder.

**FIGURE. 2:**
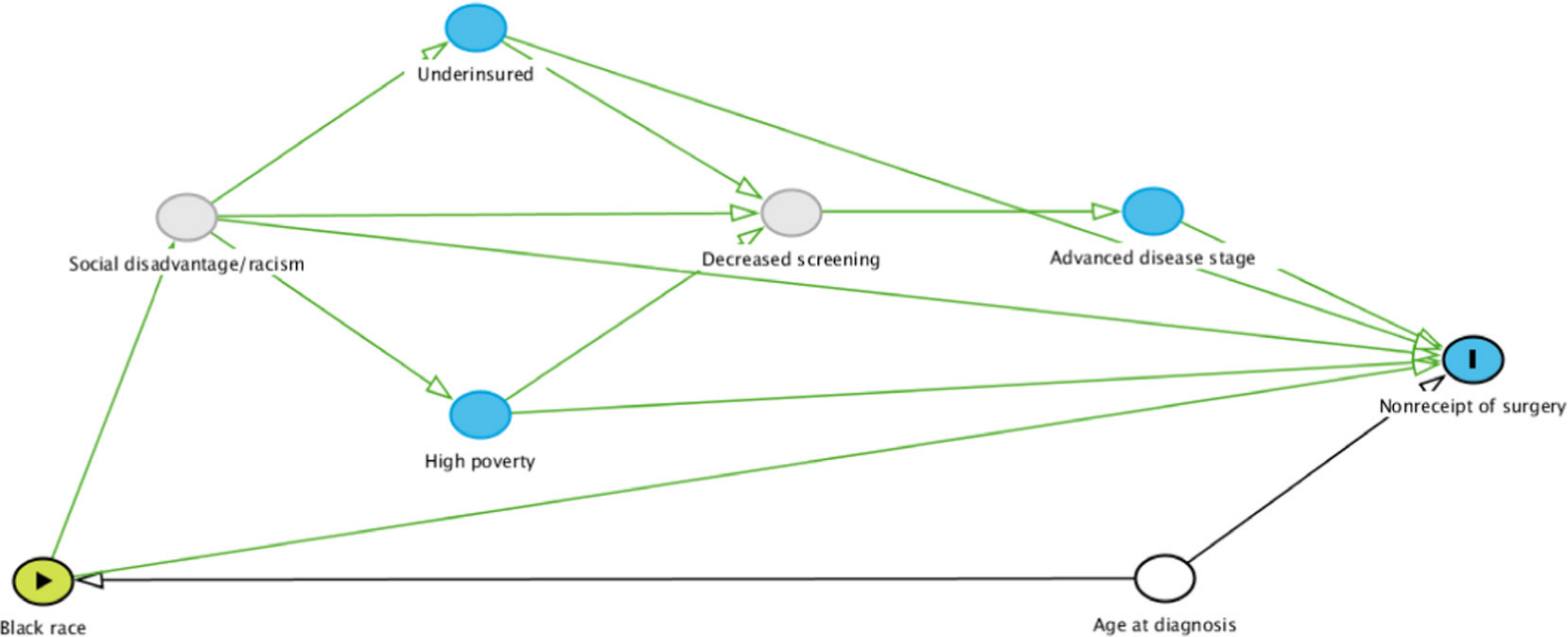
Directed acyclic graph of racial disparity in nonreceipt of surgery. Hypothesized mediators include social disadvantage/racism, underinsurance, high neighborhood poverty, decreased screening, and advanced disease stage at diagnosis. Gray mediators represent unmeasured data. Age at diagnosis is considered a confounder.

In this study, the focus was on proximal mediators and their impact on more distal mediators (the outcomes of interest in this study) of the racial disparity in breast cancer survival. As demonstrated in Figure 1, it is hypothesized that Black women diagnosed with breast cancer in this study were more likely to experience social disadvantage/racism which increased the likelihood of living in high‐poverty areas and having Medicaid/being uninsured. These mediators then decreased the likelihood of receiving breast cancer screening which correspondingly increased the risk for advanced disease stage at diagnosis. As demonstrated in Figure 2, it is also hypothesized that these factors could explain the decreased percentage of Black women undergoing surgery as treatment for their disease. In addition, more advanced disease stage at diagnosis is associated with a decreased likelihood of receiving surgery for breast cancer (with a greater effect in Black women), and surgery is not standard of care for women with metastatic disease.^18^ Therefore, disease stage was also treated as a mediator for nonreceipt of surgery.

### 2.1 Statistical analysis

Characteristics of the women in this study according to race are presented as frequencies and percentages. Log‐binomial regression was used to examine the magnitude of the age‐adjusted relative (prevalence ratio, PR) and absolute (prevalence difference, PD) racial disparity in advanced disease stage at diagnosis and nonreceipt of surgery with 95% confidence intervals (CIs). Population‐level percentages of each outcome by race were obtained by least‐squares means from the PD model.

The multiple mediation approach described by Yu et al.^19^ was used to evaluate mediation/confounding of the race effect on the two outcomes of interest. This method produces log odds ratios (ORs) for the average total, direct, and indirect effects. The average direct effect reflects the change in the log odds ratio for Black women compared to White women, independent of mediator pathways and confounders included in the model. The average indirect effect is the effect of Black race on the outcome through the mediator or due to confounding. Indirect effects of cofounders (e.g., age) can explain the effect of Black race on the outcome but are not involved in the causal pathway. The relative percentage of the average total race effect that is explained by direct and indirect effects can be obtained by dividing parameter estimates by the total effect. However, additive indirect effects may not equal the total indirect effect due to correlations and overlapping mediation effects between mediators.^19^ Statistical significance was defined as *p* < 0.05. All statistical analyses were performed using SAS (version 9.4; SAS Institute) and the mma package in R (version 4.1.1). The DAGs in this study were created with Dagitty.

## 3 RESULTS

As referenced in our earlier article, characteristics of the study population are displayed in Table 1.^11^ There were 101,872 women available for analysis, with 87% White and 13% Black. Black women were more likely to: be younger at diagnosis (≤ 64 years of age: 71.7% vs. 52.6%), have Medicaid or to be uninsured (23.4% vs. 7.7%), and reside in high‐poverty areas (44.3% vs. 11.8%).

**TABLE 1:** Demographic and clinical characteristics of Black and White women diagnosed with breast cancer in Florida, 2004–2015 (*n* = 101,872).

Characteristic	Black		White	
	*n*	%	*n*	%
Study population	13,255	13.0	88,617	87.0
Age at diagnosis				
18–29	176	1.4	282	0.3
30–39	1069	8.6	2744	3.3
40–49	2735	22.0	11,861	14.3
50–64	4986	40.1	29,154	35.0
65–74	2118	17.1	21,472	25.8
75–84	1036	8.3	13,594	16.4
≥ 85	310	2.5	4111	4.9
Insurance status				
Private	6587	49.7	41,829	47.2
Medicaid	2119	16.0	4448	5.0
Medicare	2988	22.5	37,031	41.8
Military	247	1.9	1629	1.8
Indian Health Service	129	1.0	135	0.2
Uninsured	978	7.4	2409	2.7
Unknown	207	1.6	1136	1.3
Neighborhood poverty				
Low	816	6.2	14,476	16.3
Lower‐middle	1751	13.2	29,689	33.5
Upper‐middle	4822	36.4	33,971	38.3
High	5866	44.3	10,481	11.8
AJCC disease stage				
Stage I	4310	34.7	43,214	51.9
Stage II	4729	38.0	26,524	31.9
Stage III	2376	19.1	9679	11.6
Stage IV	1015	8.2	3801	4.6
Surgery				
Yes	9897	79.6	75,833	91.1
No	2533	20.4	7385	8.9

PR and PD for the association of race with advanced disease stage at diagnosis and nonreceipt of surgery are provided in Table 2. Black women were 55% more likely (PR, 1.55; 95% CI, 1.50–1.60) to have advanced disease stage on the relative scale and 9.4% more likely (PD, 9.4%; 95% CI, 8.6–10.2%) on the absolute scale. For nonreceipt of surgery, Black women experienced a nearly twofold increased relative likelihood (PR, 1.97; 95% CI, 1.90–2.04) and a 12.3% increased absolute prevalence (95% CI, 11.5–13.1%).

**TABLE 2:** Adjusted^a^ prevalence ratios and prevalence differences for advanced disease stage at diagnosis and nonreceipt of surgery by race

	Advanced disease stage (%)	PR	95% CI	PD%	95% CI
Race					
White	16.0%	Ref.		Ref.	
Black	25.4%	1.55^b^	1.50‐1.60	9.4^b^	8.6‐10.2

	Nonreceipt of surgery (%)	PR	95% CI	PD%	95% CI
Race					
White	11.9%	Ref.		Ref.	
Black	24.2%	1.97^b^	1.90‐2.04	12.3^b^	11.5‐13.1

Abbreviations: CI, confidence interval; PD, prevalence difference; PR, prevalence ratio; Ref., reference group.

^a^ Adjusted for age at diagnosis.

^b^ *p* < 0.001.

The results of the multiple mediation analysis are displayed in Table 3. For advanced disease stage at diagnosis, having Medicaid/being uninsured explained 17.6% of the racial disparity while neighborhood poverty explained 5.3%. Confounding by age explained 12.8% of the racial disparity. The portion of the race effect that was unexplained by mediation/confounding was 64.3%. For nonreceipt of surgery, insurance status explained 6.8% of the relative disparity with neighborhood poverty explaining 3.2%. Disease stage at diagnosis mediated a large portion (31.3%) of the effect of Black race on nonreceipt of surgery while confounding by age explained 6.8%. The remaining 52.1% of the race effect was unexplained by these mediating/confounding factors.

**TABLE 3:** Log odds ratios, 95% confidence intervals, and % relative effect for average direct, indirect, and total effects of Black race on advanced disease stage at diagnosis and nonreceipt of surgery.

Effects	Advanced disease stage at diagnosis			
	Log OR	95% CI	Relative % of total effect	95% CI
Black (direct effect)	0.429^a^	0.393, 0.465	64.3^a^	61.2, 67.5
Medicaid/uninsured	0.117^a^	0.115, 0.119	17.6^a^	17.0, 18.3
High neighborhood poverty	0.035^a^	0.024, 0.046	5.3^a^	3.4, 7.2
Joint (insurance and poverty)	0.152^a^	0.141, 0.162	22.8^a^	20.5, 25.2
Age	0.085^a^	0.081, 0.089	12.8^a^	11.9, 13.7
Total effect	0.666^a^	0.642, 0.690		

Effects	Nonreceipt of surgery			
	Log OR	95% CI	Relative % of total effect	95% CI
Black (direct effect)	0.580^a^	0.547, 0.614	52.1^a^	50.1, 54.0
Insurance status	0.076^a^	0.071, 0.080	6.8^a^	6.5, 7.1
High neighborhood poverty	0.036^a^	0.026, 0.045	3.2^a^	2.3, 4.1
Joint (insurance and poverty)	0.111^a^	0.103, 0.120	10.0^a^	9.1, 10.9
Age	0.075^a^	0.072, 0.079	6.8^a^	6.4, 7.2
Disease stage	0.348^a^	0.341, 0.355	31.3^a^	30.5, 32.0
Total effect	1.114^a^	1.089, 1.139		

Abbreviations: CI, confidence interval; OR, odds ratio.

^a^ *p* < 0.001.

Post hoc multiple mediation analyses were conducted with the data stratified by age. These results are provided as supplemental content (Tables S1 and S2). In summary, for advanced disease stage at diagnosis (Table S1), insurance status played a significantly greater mediating role for Black women <65 years of age. Neighborhood poverty played a negligible role for women 18–39 years of age and had a similar impact for women 40–64 and ≥ 65 years of age. For nonreceipt of surgery (Table S2), insurance status was slightly less important for women ≥65 years of age while neighborhood poverty played a slightly greater mediating role for women 18–39 years of age.

## 4 DISCUSSION

This study was conducted to better understand how race is associated with the intermediate breast cancer outcomes of advanced disease stage at diagnosis and nonreceipt of surgery, the two most important mediators of the racial disparity in breast cancer survival for this population of women.^11^ Although this cohort of women was diagnosed relatively recently, medium to large racial disparities were present for both of these outcomes. The multiple mediation analysis revealed that, although insurance status and, to a lesser degree, neighborhood poverty, were significant mediators of the race effect for both advanced disease stage at diagnosis and nonreceipt of surgery, 64.3% and 52.1% of the race effect, respectively, were unexplained by the mediators/confounders evaluated in this study. The importance of insurance status and neighborhood poverty in mediating the race effect for these two intermediate outcomes was less than expected based on our a priori hypotheses. These results add to the scant literature on the use of mediation analysis to gain better insight into the potential causes of Black/White disparities in breast cancer outcomes. In the paragraphs that follow, we provide context to our results by comparing with similar studies and provide possible explanations for the lower‐than‐expected impact of insurance status and neighborhood poverty on these two intermediate outcomes.

Several early studies demonstrated that minority women and those of lower socioeconomic status (SES) are less likely to receive breast cancer screening via mammography which results in more advanced disease stage at diagnosis and poorer survival.^20^, ^21^, ^22^ These early studies and the recognition of the widening gap in breast cancer mortality by race led to widespread campaigns to reverse these trends.^23^ An assessment of national and Florida survey data corresponding to the years of diagnosis included in this study reveals no breast cancer screening disparities by race.^24^, ^25^ However, validation studies have reported that women overreport mammography utilization and that overreporting is greater in Black women and women of lower SES.^26^, ^27^ In addition, a meta‐analysis conducted for the years coinciding with the current study demonstrated that Black women had a 13% decreased odds of breast cancer screening over the time period of diagnosis in the current study.^28^ Furthermore, Molina et al.^29^ reported that of women diagnosed with breast cancer, Black women were less likely to be diagnosed as a result of screening. These results demonstrate that survey data of mammography prevalence are biased and that screening disparities are still present. Thus, despite numerous initiatives over several decades, Black women still have a lower likelihood of breast cancer screening.

Many historical as well as several recent studies have demonstrated that Black women with breast cancer have an increased likelihood of advanced disease stage at diagnosis compared to White women.^15^, ^30^, ^31^, ^32^, ^33^ In a recent study, Primm et al.^31^ investigated the odds of early‐stage (I–IIIA) disease for women with breast cancer in the Surveillance, Epidemiology, and End Results (SEER) database, diagnosed between 2000 and 2017. These investigators reported that, although the absolute Black/White disparity in early‐stage diagnosis decreased over the years of the study, overall, Black women had a 34% decreased odds of being diagnosed with early‐stage disease after adjustment for demographic factors including county‐level median household income. Lipscomb et al.^34^ reported an unadjusted OR = 1.81 for Black/White advanced disease at diagnosis which decreased to OR = 1.16 after adjustment for method of detection (i.e., mammography), tumor‐related characteristics, and demographic factors including census‐tract poverty level, census‐tract education, and individual insurance status. Epidemiologic studies which compare measures of association pre‐ and post‐adjustment demonstrate that including mediators in statistical models will attenuate the race effect, but they are not a formal mediation analysis which can quantify the relative amount of the disparity explained by each mediator.

To our knowledge, the only study that has investigated mediation of the race effect on the outcome of advanced disease stage at diagnosis was by Ko et al.^15^ These investigators evaluated insurance status (Medicaid/uninsured vs. private) as a mediator of the Black/White disparity in advanced stage of disease at diagnosis (disease Stage III vs. I/II) for women 40–64 years of age with invasive, nonmetastatic disease in the SEER database. Compared to our findings, these researchers reported a larger mediating impact of health insurance status (45% vs. 18%). Explanations for the discrepancy between our results with these investigators include the type of mediation analysis (multiple mediation vs. single mediator effects), a different study population (Florida vs. SEER), and, in the current study, inclusion of all women ≥18 years of age and those with metastatic (Stage IV) disease. To evaluate whether differences in age and disease stage could explain the discrepancy in results concerning the importance of insurance status as a mediator, we conducted a post hoc analysis and limited our study population to women 40–64 years of age with Stage I–III disease. The same multiple mediation analysis for advanced disease at diagnosis described in this study was then performed with this restricted dataset. With these additional exclusions, the importance of insurance as a mediator of the race effect on advanced disease stage only slightly increased (19.3% vs. 17.6%). These differing results call for further study.

Our result concerning the increased likelihood of Black women with breast cancer to not receive surgery as treatment was also found in prior studies.^7^, ^32^, ^35^, ^36^ To our knowledge, no other studies have investigated potential mediators of the race effect on disparities in nonreceipt of surgery. In this study, the mediating impact of insurance status and neighborhood poverty were even less impactful for nonreceipt of surgery. Results from other large epidemiologic studies have reported that lower income, lower education, public health insurance, metropolitan residence, advanced disease stage, older age, comorbid conditions, healthcare access, and patient choice are associated with a decreased likelihood of receiving surgery.^36^, ^37^ The relationship between socioeconomic factors and nonreceipt of surgery reported in prior studies led to our hypothesis that these factors could potentially explain a sizable portion of the racial disparity in this outcome for women with breast cancer. These factors did explain 10% of the disparity, which is not insignificant, but better understanding of the other mediators driving the racial disparity in nonreceipt of surgery is needed.

Evidence of other factors responsible for the racial disparity in advanced disease stage at diagnosis and nonreceipt of surgery comes from other study designs (survey/qualitative/prospective cohort), assessing characteristics that are usually absent in epidemiologic studies using cancer registry data. As others have noted, inclusion of additional and refinement of existing sociodemographic characteristics of patients in cancer registries and development of reporting standards would yield greater insight into the causes of cancer health disparities.^38^ Barriers to preventive care and cancer screening for Black women cited by prior studies include: difficulty finding time for screening within the context of other responsibilities (e.g., caretaking, employment), lack of knowledge of screening benefits, cultural beliefs, not having a regular source of care, distrust of the healthcare system, discrimination/racism, fear/fatalism, differing screening recommendations by guideline‐issuing groups, economic challenges, health utilization, physician recommendation, transportation and travel distance barriers, and pain associated with mammogram.^39^, ^40^, ^41^, ^42^ Many of these barriers are also present for receipt of surgery and other treatment modalities including: mistrust of the healthcare system and providers, the patient–physician relationship, psychosocial characteristics, discrimination/racism, concerns about the quality and costs of care, transportation barriers, treatment‐related side effects, and the effect on romantic relationships.^43^, ^44^, ^45^, ^46^, ^47^, ^48^ In addition, Black women with breast cancer are more likely to experience fragmented cancer care from lower quality facilities.^49^, ^50^ As noted by Polacek et al.,^51^ treatment decision‐making involves a complex interplay of multifactorial elements. Multilevel interventions which focus on addressing barriers at the patient, provider, and health system/policy level may be the best approach to reducing impediments to high‐quality cancer care.^52^, ^53^, ^54^

There are several study limitations to be considered. As this is a cross‐sectional study, insurance status and neighborhood poverty were determined at the time of diagnosis. An assumption was that the measurement of these factors at the time of diagnosis was representative of the time prior to diagnosis, which influenced screening decisions and thus, disease stage at diagnosis. However, if values for these mediators at diagnosis did not represent the prediagnosis period, there could have been some degree of misclassification. A second limitation is that the DAGs used in this study are simplified versions, depicting hypothesized paths of the race effect for the mediators examined in this study. The direct effect captures the effect of race that is independent of mediator pathways, adjusted for confounding factors. As noted, other factors of patients, providers, and the healthcare system may mediate the racial disparity in these two outcomes, but these characteristics are typically not measured in epidemiologic studies. Another limitation is that other than Medicaid enrollment which is a proxy for individual/household poverty, we did not have an individual/household measure of income. Neighborhood poverty may reflect individual/household poverty but may also encompass lower healthcare resources for women living in these areas and greater transportation challenges. Furthermore, the “no confounding” assumptions for mediation analysis apply to numerous levels of exposure, mediator, and outcome and cannot be verified.^55^ Thus, we cannot conclude that the associations reported in this study for total, direct, and indirect effects represent causal relationships. Another limitation of the study is related to generalizability in that the relationships reported may not be representative of other racial/ethnic groups as we restricted our study to Black/White women. Finally, although the Florida Cancer Data System has received Gold Certification from the North American Association of Central Cancer Registries, we either lacked or had incomplete data on other factors that may also play a mediating role including body mass index, comorbidities, and alcohol/tobacco use.^56^

Despite some progress that has occurred in reducing racial disparities in breast cancer outcomes, in this study, Black women were significantly more likely to be diagnosed with advanced disease stage at diagnosis and to not receive surgery for their disease. We found that insurance status was more important than neighborhood poverty in explaining the race effect on both outcomes, and the mediating role of these SES‐related factors was greater for advanced disease stage at diagnosis than nonreceipt of surgery. However, the majority of the race effect on these two outcomes remained unexplained after accounting for examined mediators/confounders. Additional barriers that could explain the racial disparity in these two outcomes have been reported in other study designs, and these factors are usually not available in cancer epidemiologic studies using registry data. Multilevel interventions that address these additional barriers to breast cancer screening and receipt of high‐quality treatment are needed to reach the goal of racial equity in breast cancer outcomes.

## AUTHOR CONTRIBUTIONS

**Robert B. Hines:** Conceptualization (lead); data curation (lead); formal analysis (lead); funding acquisition (lead); investigation (lead); methodology (lead); project administration (lead); writing – original draft (lead); writing – review and editing (equal). **Xiang Zhu:** Data curation (equal); formal analysis (equal); methodology (equal); software (equal); writing – review and editing (equal). **Eunkyung Lee:** Formal analysis (equal); investigation (equal); methodology (equal); writing – review and editing (equal). **Bradley Eames:** Investigation (equal); writing – review and editing (equal). **Karolina Chmielewska:** Investigation (equal); writing – review and editing (equal). **Asal M. Johnson:** Conceptualization (equal); investigation (equal); methodology (equal); writing – review and editing (equal).

## FUNDING INFORMATION

This work was supported by a research grant from the Florida Breast Cancer Foundation.

## CONFLICT OF INTEREST STATEMENT

All authors declare no conflicts of interest.

## ETHICS STATEMENT

This study was approved by the IRBs of the FDOH and UCF.

## Supporting information


Table S1.
Table S2.

## Data Availability

The data used for this research are available from the Florida Cancer Data System of the Florida Department of Health (FDOH). We are willing to share the data with outside researchers provided all permissions to do so have been met in accordance with state law.
