# Supplementary material for: Health insurance and neighborhood poverty as mediators of racial disparities in advanced disease stage at diagnosis and nonreceipt of surgery for women with breast cancer
Source: Cancer Med. 2023 Jun 6;12(14):15414–23. doi: 10.1002/cam4.6127 (PMC10417299; doi:10.1002/cam4.6127)
Supplement: Supplementary file 1 — Table S1. Table S2. [file CAM4-12-15414-s001.docx]

| **Supplementary Table 1.** Log odds ratios, 95% confidence Intervals, and % relative effect for average direct, indirect, and total effects of Black race on advanced disease stage at diagnosis, stratified by age | | | | | | | | |
| --- | --- | --- | --- | --- | --- | --- | --- | --- |
|  |  |  | **Advanced Disease Stage at Diagnosis** | | | | | |
| **Age** | **n** | **Effects** | **Log OR** | **95% CI** | ***p*** | **Relative % of Total Effect** | **95% CI** | ***p*** |
| 18-39 | 4,432 | Black (direct effect) | 0.336 | 0.284, 0.388 | <0.001 | 74.7 | 68.3, 81.1 | <0.001 |
|  |  | Medicaid/uninsured | 0.099 | 0.072, 0.126 | 0.001 | 21.7 | 18.6, 24.9 | <0.001 |
|  |  | High neighborhood poverty | 0.004 | -0.033, 0.040 | 0.826 | 0.5 | -8.6, 9.7 | 0.910 |
|  |  | Joint (Insurance and poverty) | 0.103 | 0.064, 0.142 | 0.005 | 22.4 | 15.3, 29.4 | 0.002 |
|  |  | Age | 0.013 | 0.009, 0.017 | 0.002 | 3.0 | 2.0, 3.9 | 0.003 |
|  |  | Total effect | 0.452 | 0.374, 0.529 | <0.001 |  |  |  |
| 40-64 | 51,764 | Black (direct effect) | 0.361 | 0.340, 0.383 | <0.001 | 68.4 | 66.1, 70.6 | <0.001 |
|  |  | Medicaid/uninsured | 0.121 | 0.118, 0.123 | <0.001 | 22.8 | 22.1, 23.6 | <0.001 |
|  |  | High neighborhood poverty | 0.035 | 0.026, 0.045 | 0.001 | 6.7 | 4.8, 8.6 | 0.001 |
|  |  | Joint (Insurance and poverty) | 0.155 | 0.146, 0.164 | <0.001 | 29.4 | 27.1, 31.6 | <0.001 |
|  |  | Age | 0.011 | 0.010, 0.012 | <0.001 | 2.1 | 1.9, 2.4 | <0.001 |
|  |  | Total effect | 0.528 | 0.514, 0.543 | <0.001 |  |  |  |
| 65+ | 45,666 | Black (direct effect) | 0.539 | 0.495, 0.583 | <0.001 | 83.7 | 81.2, 86.2 | <0.001 |
|  |  | Medicaid/uninsured | 0.061 | 0.056, 0.065 | <0.001 | 9.4 | 8.8, 10.1 | <0.001 |
|  |  | High neighborhood poverty | 0.047 | 0.033, 0.060 | 0.002 | 7.3 | 5.0, 9.5 | 0.002 |
|  |  | Joint (Insurance and poverty) | 0.107 | 0.093, 0.122 | <0.001 | 16.7 | 14.3, 19.2 | <0.001 |
|  |  | Age | -0.003 | -0.005, -0.002 | 0.029 | -0.5 | -0.7, -0.3 | 0.005 |
|  |  | Total effect | 0.644 | 0.601, 0.687 | <0.001 |  |  |  |
| Abbreviations: OR, odds ratio; CI, confidence interval. | | | | | | | | |

| **Supplementary Table 2.** Log odds ratios, 95% confidence Intervals, and % relative effect for average direct, indirect, and total effects of Black race on advanced disease stage at diagnosis, stratified by age | | | | | | | | |
| --- | --- | --- | --- | --- | --- | --- | --- | --- |
|  |  |  | **Nonreceipt of Surgery** | | | | | |
| **Age** | **n** | **Effects** | **Log OR** | **95% CI** | ***p*** | **Relative % of Total Effect** | **95% CI** | ***p*** |
| 18-39 | 4,432 | Black (direct effect) | 0.422 | 0.273, 0.571 | 0.003 | 58.4 | 48.5, 68.3 | <0.001 |
|  |  | Medicaid/uninsured | 0.049 | 0.032, 0.066 | 0.003 | 7.3 | 4.0, 10.6 | 0.009 |
|  |  | High neighborhood poverty | 0.037 | 0.009, 0.065 | 0.046 | 5.6 | 1.1, 10.0 | 0.050 |
|  |  | Joint (Insurance and poverty) | 0.087 | 0.069, 0.104 | <0.001 | 13.0 | 8.5, 17.4 | 0.003 |
|  |  | Age | 0.007 | 0.005, 0.009 | 0.001 | 1.0 | 0.6, 1.3 | 0.002 |
|  |  | Disease stage | 0.198 | 0.164, 0.233 | <0.001 | 28.8 | 22.4, 35.1 | <0.001 |
|  |  | Total effect | 0.706 | 0.568, 0.844 | <0.001 |  |  |  |
| 40-64 | 51,764 | Black (direct effect) | 0.635 | 0.618, 0.653 | <0.001 | 60.3 | 59.3, 61.3 | <0.001 |
|  |  | Medicaid/uninsured | 0.076 | 0.070, 0.081 | <0.001 | 7.2 | 6.8, 7.5 | <0.001 |
|  |  | High neighborhood poverty | 0.024 | 0.017, 0.030 | 0.002 | 2.3 | 1.6, 2.9 | 0.001 |
|  |  | Joint (Insurance and poverty) | 0.100 | 0.091, 0.108 | <0.001 | 9.5 | 8.8, 10.1 | <0.001 |
|  |  | Age | 0.007 | 0.005, 0.009 | 0.001 | 0.7 | 0.5, 0.9 | 0.001 |
|  |  | Disease stage | 0.312 | 0.299, 0.325 | <0.001 | 29.6 | 28.5, 30.7 | <0.001 |
|  |  | Total effect | 1.054 | 1.025, 1.084 | <0.001 |  |  |  |
| 65+ | 45,666 | Black (direct effect) | 0.610 | 0.544, 0.677 | <0.001 | 62.5 | 60.2, 64.9 | <0.001 |
|  |  | Medicaid/uninsured | 0.051 | 0.042, 0.059 | <0.001 | 5.3 | 4.0, 6.6 | <0.001 |
|  |  | High neighborhood poverty | 0.035 | 0.015, 0.054 | 0.017 | 3.4 | 1.7, 5.2 | 0.013 |
|  |  | Joint (Insurance and poverty) | 0.086 | 0.069, 0.103 | <0.001 | 8.8 | 7.5, 10.0 | <0.001 |
|  |  | Age | -0.001 | -0.007, 0.004 | 0.728 | -0.1 | -0.7, 0.4 | 0.728 |
|  |  | Disease stage | 0.283 | 0.266, 0.299 | <0.001 | 29.1 | 27.2, 31.0 | <0.001 |
|  |  | Total effect | 0.644 | 0.601, 0.687 | <0.001 |  |  |  |
| Abbreviations: OR, odds ratio; CI, confidence interval. | | | | | | | | |
